# Biomass Extraction Using Non-Chlorinated Solvents for Biocompatibility Improvement of Polyhydroxyalkanoates

**DOI:** 10.3390/polym10070731

**Published:** 2018-07-03

**Authors:** Guozhan Jiang, Brian Johnston, David E. Townrow, Iza Radecka, Martin Koller, Paweł Chaber, Grażyna Adamus, Marek Kowalczuk

**Affiliations:** 1 School of Biology Chemistry and Forensic Science, University of Wolverhampton, Wolverhampton WV11LY, UK; guozhan.jiang@cranfield.ac.uk (G.J.); b.johnston@wlv.ac.uk (B.J.); D.Townrow@wlv.ac.uk (D.E.T.); 2 C/o Institute of Chemistry, Office of Research Management and Service, University of Graz, NAWI Graz, Heinrichstrasse 28/III, 8010 Graz, Austria; martin.koller@uni-graz.at; 3 Centre of Polymer and Carbon Materials, Polish Academy of Sciences, M. Curie-Skłodowskiej 34, 41-819 Zabrze, Poland; pchaber@cmpw-pan.edu.pl (P.C.); grazyna.adamus@cmpw-pan.edu.pl (G.A.)

**Keywords:** cyclohexanone, γ-butyrolactone, chloroform, extraction, polyhydroxyalkanoates, PHB

## Abstract

An economically viable method to extract polyhydroxyalkanoates (PHAs) from cells is desirable for this biodegradable polymer of potential biomedical applications. In this work, two non-chlorinated solvents, cyclohexanone and γ-butyrolactone, were examined for extracting PHA produced by the bacterial strain *Cupriavidus necator* H16 cultivated on vegetable oil as a sole carbon source. The PHA produced was determined as a poly(3-hydroxybutyrate) (PHB) homopolyester. The extraction kinetics of the two solvents was determined using gel permeation chromatography (GPC). When cyclohexanone was used as the extraction solvent at 120 °C in 3 min, 95% of the PHB was recovered from the cells with a similar purity to that extracted using chloroform. With a decrease in temperature, the recovery yield decreased. At the same temperatures, the recovery yield of γ-butyrolactone was significantly lower. The effect of the two solvents on the quality of the extracted PHB was also examined using GPC and elemental analysis. The molar mass and dispersity of the obtained polymer were similar to that extracted using chloroform, while the nitrogen content of the PHB extracted using the two new solvents was slightly higher. In a nutshell, cyclohexanone in particular was identified as an expedient candidate to efficiently drive novel, sustainable PHA extraction processes.

## 1. Introduction

The search for biomaterials that are able to provide the best performance with an appropriate host response in medical devices has been based upon the understanding of all of the interactions within the biocompatibility phenomena [1]. Biomass-derived polyhydroxyalkanoates (PHA) constitute biomaterials of potential value for medical applications [2]. The structure of the deposited PHA granules consists of a polyester core surrounded by an organic shell composed of phospholipids, structural proteins, regulating proteins, polyester synthase, and depolymerase [3]. In order to eliminate microbial components (cell debris or metabolites) from crude PHA, the biopolymer has to be carefully purified before it is processed. In particular, bacterial endotoxins (heat-resistant lipopolysaccharides), which are synthesized primarily by Gram-negative microbes, and protein traces need to be efficiently removed [4]. Recently, the facile method for PHA purity evaluation versus protein residues by elemental analysis has been reported by some studies [5].

The existing recovery processes can be divided into two categories: disruption of the non-PHA cellular materials (NPCM) (including nucleic acids, lipids and phospholipids, peptidoglycan, proteinaceous materials including glycoproteins and, in some cases, lipopolysaccharides and other carbohydrates), followed by the release of the intact PHA granules from inside; and direct solvent extraction from within the cells. In the former method, the cell walls can be disintegrated using chemical, enzymatic, or mechanical means [6]. In a chemical digestion of the non-PHA cellular materials, the chemicals used are aqueous sodium hypochlorite solution (NaClO) [7], aqueous surfactant solutions [8], or a combination of surfactants and NaClO [8,9]. Enzymatic digestion of NPCM was first invented by ICI in the early 1990s where the NPCM was digested by a cocktail of proteolytic enzymes (proteases) in one stage or several stages [10]. Both the polymer yield and purity are low for these types of methods. Further purification is usually carried out by solvent extraction of the obtained PHAs. Some green solvents have been examined for disrupting cell walls. 1-ethyl-3-methylimidazolium methylphosphonate, an ionic liquid, has been used to disrupt cyanobacteria cell walls to extract the PHAs inside [11]. Supercritical CO_2_ (scCO_2_) has also been used to disrupt *Ralstonia eutropha* (today: *Cupriavidus necator*) biomass for the recovery of PHAs at 200 atm and 40 °C for 100 min, and 89% of PHA was recovered from the biomass [12]. This process was only recently optimized by using both scCO_2_ and “CO_2_ expanded ethanol”, the latter allowing the recovery of highly pure PHA at a reduced temperature when compared to the use of scCO_2_ alone. However, the ionic liquid per se is currently expensive, and scCO_2_, although it does not leave behind any residues as all the other solvents do, is expensive in terms of equipment investment. For these reasons, their application to recover PHAs is not economically acceptable at present. Moreover, the PHAs undergo significant degradation after disrupting non-PHA cellular materials using these types of methods. For example, the molar mass of PHAs has a reduction of approximately 50% after treatment with NaClO to disrupt the cell walls [7]. Only recently, a rather exotic, biological method to generate intact PHA granules without affecting the native structure of the biopolyester was reported, which is based on the digestion of PHA-rich bacterial biomass by the mealworm *Tenebrio molitor*; as a product of digestion, PHA granules of remarkable purity exceeding 90% were obtained, which could be further purified using water and NaOH; of course, this approach is still in its infancy and it is doubtful regarding its suitability for large scale implementation [13].

In solvent extraction, a PHA solvent is used to dissolve PHA inside the cells, which is then precipitated from the solution using a PHA anti-solvent after removing the cell wall debris from the solution. Before extraction, a cell breakage step should be adopted to facilitate the extraction. In most cases, heating at above 100 °C is enough to weaken the cells of the best described PHA production strains such as *C. necator* [14]. Generally, the efforts needed to break the cell walls strongly depend on the type of production strain; whereas some strains like recombinant *Eschericha coli* (no natural PHA producers) easily burst, Gram-positive cells are typically more recalcitrant towards cell disintegration. In order to facilitate separation of the cell debris from the resultant PHA solution, the PHA concentration should be less than 5% by weight as the solution tends to be very viscous, rendering subsequent separation of the cell debris difficult.

There are not many solvents that can dissolve rather hydrophobic PHAs, especially those with a short chain length, e.g., poly(3-hydroxybutyrate) (PHB); this is especially valid for solvents of considerable polarity. Therefore, direct PHA extraction often resorts to chlorinated hydrocarbons such as chloroform, dichloromethane, or 1,2-dichloroethane [14], by which a highly pure PHA and high PHA recovery yield can be achieved. However, they have a severe toxicity and high environmental impact; their use clearly counteracts the sustainability principles of PHA manufacturing [15]. Hence, less toxic non-halogenated solvents have been investigated to develop solvent-based recovery systems. Many non-halogenated solvents have been mentioned in patents [16,17,18] such as cyclic carbonic esters, methyl ethyl ketone, cyclohexanone (CYC), and γ-butyrolactone (GBL). Among these potential solvents, only a few have been investigated in detail. Koller et al. [19] developed a high pressure unit to use acetone at 120 °C (above the solvent’s boiling point) and a pressure of 7 bar to dissolve a short chain length PHA, poly(3-hydroxybutyrate-*co*-21.8%-3-hydroxyvalerate-*co*-5.14%-4-hydroxybutyrate), with a recovery yield of 98.9% and a purity of 99.0%; precipitation of the PHA from acetone simply occurs during cooling down to room temperature. Yang et al. [20] investigated the use of methyl ethyl ketone as a solvent to extract poly(3-hydroxybutyrate-*co*-3-hydroxyvalerate). The extraction was conducted at 100 °C for 5 min to achieve a recovery yield of 93% and a purity of 91%. Since methyl ethyl ketone has a boiling point of only 79.6 °C, this extraction was also conducted using pressurized equipment. Rosengart et al. [21] investigated anisole and CYC as the solvents to extract PHB from *Burkholderia sacchari* cells. Polymer recovery yields of 97% and 93% were obtained, respectively, at 120–130 °C for about 15–30 min using a cell/solvent ratio of 1.5% (*w*/*v*). Maximum polymer purities using these experimental conditions were 98% for both solvents.

In the above solvent-based recovery systems, kinetics of the recovery was scarcely investigated, which is important for the design of emerging application areas. In this work, the kinetics of the extraction of PHAs from the cells was investigated via the use of gel permeation chromatography (GPC). This method is universal for other solvent systems. For this purpose, two solvents, CYC and GBL, were selected. The two solvents in this work have high boiling points (156 °C and 204 °C, respectively), which will be an advantage for the extraction to be conducted at atmospheric pressure but high temperature. The former was investigated primarily in terms of health and safety for the extraction of PHAs [21], while the latter has not been studied in detail in the open literature.

## 2. Materials and Methods

### 2.1. Synthesis

The PHA contained in biomass was produced via a two-stage fermentation process with strain *C. necator* H16 with vegetable oil as the sole carbon source in a 5 L batch fermenter (Electrolab FerMac 310). The starter medium contained 30 g/L of tryptic soy broth (TSB). For production, a basal salt medium (BSM) with the following composition was used: 1 g/L Na_2_HPO_4_·2H_2_O, 1 g/L KH_2_PO_4_, 1 g/L (NH_4_)_2_SO_4_, 0.1 g/L MgSO_4_·7H_2_O, 0.1 g/L KNO_3_, 0.1 g/L NaCl, and 10 mL/L trace element solution. The trace element solution consisted of 2 g/L FeCl_3_, 2 g/L CaCl_2_, 2 g/L CuSO_4_·5H_2_O, 2 g/L MnSO_4_·5H_2_O, 2 g/L ZnSO_4_·5H_2_O, and 2 g/L (NH_4_)_6_Mo_7_O_24_·4H_2_O.

The starter culture was prepared by inoculation of 250 mL TSB solution in a 500 mL flask with a single colony of the strain; incubation was done at 35 °C for 24 h with a shaking rate of 150 rpm. The broth was then centrifuged to remove the clear supernatant, then the solid bacterial pellet was resuspended in 500 mL BSM; this culture was transferred into the fermenter as inoculum. The sole carbon source vegetable oil (from a local Asda supermarket, Waterloo Road, Wolverhampton, UK) was first mixed with 500 mL BSM, and then emulsified using sonication (Bandelin Electronic sonicator, Berlin, Germany). The vegetable oil/BSM mixture was then transferred into the fermenter. The total volume of the production medium was 3500 mL (including the inoculum, oil, and BSM) containing 40 g of vegetable oil in the 5 L batch fermenter.

The production was carried out for 72 h at 30 °C. The pH was automatically controlled at 7.0 ± 0.05 by adding either 2 M HCl or 2 M NaOH. Dissolved oxygen was controlled at below 4.5% of the saturation concentration of oxygen by regulating the stirrer speed and sterile air flow rate.

### 2.2. Downstream Extraction and Determination of Extraction Kinetics

After centrifugation (4500 rpm, 10 min, Sigma 6-16S, Sigma Laborzentrifugen GmbH, Osterode am Harz, Germany) and lyophilization (−40 °C, 5 mbar, 48 h, Edwards freeze dryer, Crawley, UK), the dried biomass was degreased in acetone (AR, Fisher Scientific, Loughborough, UK) with a volume/mass ratio of 20/1 at room temperature overnight under magnetic stirring [22]. The degreased dried biomass was then extracted using chloroform (AR, Fisher), CYC (AR, Fisher Scientific, Loughborough, UK), and GBL (AR, Fisher Scientific, Loughborough, UK), respectively. Chloroform was used as the control solvent for this work, acting as the “benchmark” to assess the performance of the other two solvents. Ten times the volume of methanol (AR, Fisher Scientific, Loughborough, UK) was used as the PHA anti-solvent to precipitate the dissolved PHAs from their solutions. The precipitation was performed by dropwise addition of the polymer solution into methanol under vigorous magnetic stirring. When chloroform was used as the extracting solvent, a Soxhlet extractor was used. The biomass was placed in a thimble, and the polymer was extracted into the solution in the distillation flask. When CYC or GBL was used as the extracting solvent, the biomass was placed into the solvent with a ratio of 1/50 (g/mL). The extraction was performed at 80–120 °C under vigorous stirring. After half an hour, the solution was hot filtered to remove the cell debris, and the polymer was then precipitated from the solution by adding methanol.

To determine the kinetics of the extraction, 3 mL of the solvent was heated to a predetermined temperature in a 10 mL round bottom flask. In this work, three temperature levels were used: 80, 100, and 120 °C. After the temperature was reached, 0.30 g of dried biomass was added into the hot solvent with magnetic stirring. At different time intervals, 0.1 mL of the mixture was sampled and immediately dissolved in HPLC grade chloroform for GPC analysis as follows. The concentration of PHA extracted in the solvent was determined using the area ratio of the two peaks on the GPC chromatogram, one for PHA and one for the solvent. The area ratio was calibrated using several ratios of PHA and the solvent in chloroform. The recovery yield of PHA was calculated using the following equation:

*y* = *Vd_s_x*/(*mx*_0_) (1)

where *y* is the recovery yield; *V* is the total volume of the solvent; *d_s_* is the density of the solvent; *x* is the mass fraction of the PHA in the solution measured by GPC as described in the following; *m* is the mass of the dried biomass added to the solvent; and *x*_0_ is the PHA content in the dried biomass determined by GC-MS.

### 2.3. Determination of PHA Using GC-MS

The PHA in the dried biomass and the purity of the PHA extracted were determined using GC-MS. For this purpose, the dried biomass or the extracted PHA was subjected to methanolysis in the presence of sulfuric acid according to the well-established procedure [23]. Benzoic acid was used as the internal standard. The samples were then analyzed on an Agilent 7890B GC coupled with a 5977A mass spectrometer (Santa Clara, CA, USA). The column used was DB5-MS UI with a size of 30 m × 0.25 mm × 0.25 mm. The temperatures of the injection port, interface, quadrupole, and ion source were set at 250 °C, 280 °C, 120 °C, and 250 °C, respectively. The oven temperature was programmed at an initial temperature of 40 °C and subsequently raised at a rate of 10 °C /min to 280 °C and held for 5 min. Helium carrier gas was set at a flow rate of 1.2 mL/min. Solvent delay was set at 2.5 min. The MS detector using electron impact (EI) ionization at 70 eV was operated in full scans (mass range of *m*/*z* 40 to 600).

### 2.4. Nuclear Magnetic Resonance (NMR)

The ^1^H NMR spectra of the extracted PHA were recorded on a JEOL ECX-400 (400 MHz) spectrometer (Hertfordshire, UK). The solvent for the NMR experiments was chloroform-*d*. For the ^1^H and ^13^C spectra, 16 and 1024 scans, respectively, were used. ^1^H spectrum chemical shifts were referred to CHCl_3_ (δ = 7.25 ppm), ^13^C spectrum chemical shifts to CDCl_3_ (δ = 77.00 ppm).

### 2.5. Gel Permeation Chromatography (GPC)

The kinetics of the extraction was determined using a TOSOH EcoSec HLC/GPC 8320 system GPC system (gel permeation chromatography). The GPC system was equipped with a UV and RI detector, operated at a temperature of 40 °C. The column used was a TSKgel HZM-N calibrated against monodisperse polystyrene standards ranging from 560 to 70,000 Da. The UV detector was set at a wavelength of 254 nm. Chloroform was used as the eluent at a flow rate of 0.25 mL/min. A sample size of 2 μL was injected into the system using an autosampler.

### 2.6. Elemental Analysis

The elemental composition of the extracted PHA was determined using a CHN analyzer. Elemental analysis as a measure for product purity was performed using a Perkin Elmer Analyzer 2400 as described in [5].

## 3. Results and Discussion

Biotechnological production of PHAs with *C. necator* is a well-established procedure [24]. In this work, the PHA was produced using vegetable oil as the sole carbon source in a batch fermenter for 72 h. The final dried biomass collected was 30.1 g with a PHA content of 82.3%. The conversion yield of substrate to PHA was about 0.62 g/g. About 2 g of the dried biomass was extracted using boiling chloroform under reflux in a Soxhlet extractor for 24 h. The extracted PHA was analyzed using NMR to determine the type of the PHA.

**Figure 1:**
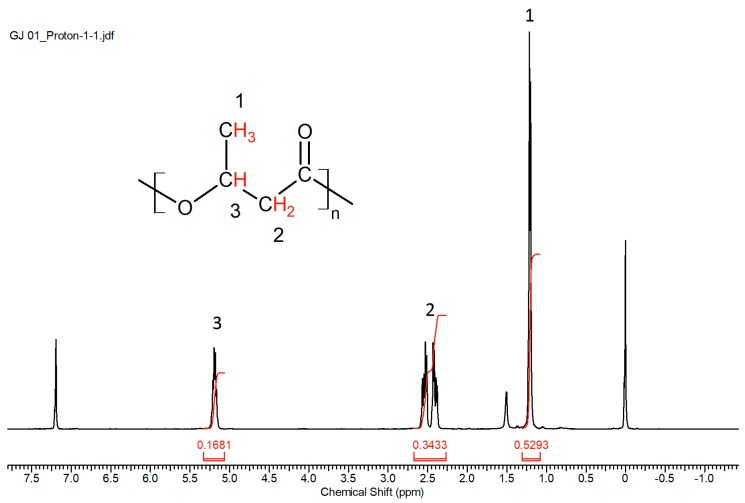
^1^H NMR spectrum of the PHA extracted using chloroform. ^1^H NMR (400 MHz, Chloroform-d) δ 5.19 (d, *J* = 6.7 Hz, 1H), 2.69–2.24 (m, 2H), 1.20 (d, *J* = 6.2 Hz, 3H).

**Figure 2:**
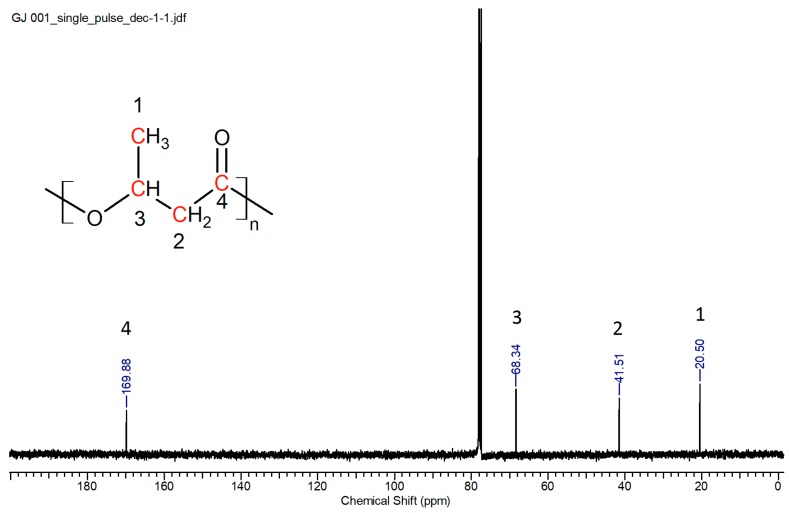
^13^C NMR spectrum of the PHA extracted using chloroform.

The ^1^H NMR spectrum of the PHA is shown in Figure 1. Peak 1 at 1.20 ppm was attributed to the methyl protons (side chain of 3-hydroxybuytrate). Peak 2 at 2.69–2.24 ppm was due to the diasterotopic protons at position 2 of the chemical structure (backbone of 3-hydroxybutyrate). Peak 3 at 5.19 ppm is due to the proton bonded to the carboxyl oxygen. The ^13^C NMR spectrum of the obtained PHA is shown in Figure 2. There were four peaks (δ = 20.5 ppm, 41.5 ppm, 68.3 ppm, and 170 ppm) in the spectrum that could be attributed to methyl carbon (side chain of 3-hydroxybuytrate), methylene carbon (backbone of 3-hydroxybutyrate), methine carbon (chiral center of 3-hydroxybutyrate), and carbonyl carbon, respectively. From both the ^1^H and ^13^C spectra, it can be confirmed that the PHA produced was primarily PHB.

**Figure 3:**
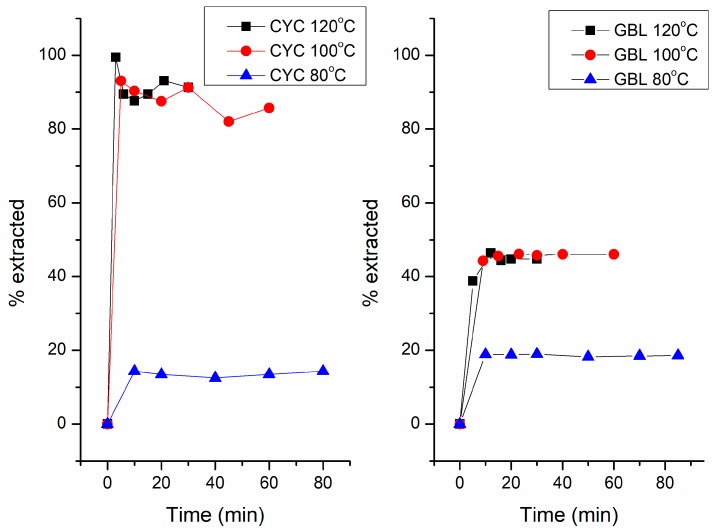
Extraction kinetics of PHA from cells using cyclohexanone (CYC, **left**) and γ-butyrolactone (GBL, **right**) at various temperatures.

After characterization of the PHA produced, extraction using CYC and GBL was performed. The effects of extraction time and temperature were studied on the recovery yield and molar mass. The PHB in the biomass was first extracted using chloroform, and characterized using GC-MS, but its extraction kinetics was not investigated due to the limitation of the methodology. Mainly the extraction kinetics of CYC and GBL were the focus of our interest. A plot of the percentage of polymer extracted from the cells against extraction time is shown in Figure 3. For each solvent, the extraction was carried out at three temperature levels: 80 °C, 100 °C, and 120 °C. The left panel of Figure 3 shows the kinetics of the extraction using CYC. At 80 °C, only 16% of the polymer was extracted regardless of how long the extraction was conducted. In our experiment, the extraction time was extended to 20 h at 80 °C. However, the recovery yield was not significantly increased, still maintaining at around 16%. When the temperature was increased to 100 °C, about 90% of the polymer was extracted within 5 min. By further increasing the extraction time, the recovery did not increase significantly. When the temperature was increased to 120 °C, about 99% of the polymer was extracted within 3 min. Further increase in extraction time did not improve the recovery yield. After kinetic measurement, the polymer was precipitated using methanol. To confirm the structure and purity of the extracted PHA, GC-MS analysis after methanolysis was performed (Section 2.3). The presence of methyl 3-hyroxybutyrate was indicated and PHB purity was 99.5%. The right panel of Figure 3 shows the kinetics of extraction using GBL. At 80 °C, only 18% of the polymer was extracted no matter how long the extraction time was extended, which is in accordance with the results obtained using CYC. In contrast to CYC, the recovery yield of PHA was only 45% at 100 °C and 120 °C; no significant difference was observed at these two temperature levels. After 20 min, the recovery yield did not increase further with the extraction time. After kinetic measurement, the polymer was precipitated using methanol. The purity of the PHB was 97.2% as measured by GC-MS.

From the above results shown in Figure 3, it is interesting to note that the extraction of PHA from the cells was quite fast, especially at higher temperatures (100 °C and 120 °C). The recovery yield depends on both temperature and solvent type, but is less dependent on extraction time. This indicates that the solubility of the solvents restricts the recovery yield, while the solubility of the solvent is again a function of temperature. It is known that the polymer occurs as an intracellular product (“carbonosomes”) [25]. The rapid dissolution of the polymer suggests that the synergistic action of the drying process and elevated temperature sufficiently weakens the bacterial cells to enable the PHB to be extracted without the necessity for any previous cell breakage step [14]. As the crystal melting point of PHB is about 120 °C [26], the rapid dissolution of the polymer may be due to the melting of the PHA crystals before dissolution in the solvents. There was a big difference in the recovery yield for the two solvents. This was probably due to the difference in the solubility of PHB in the two solvents. The difference can be illustrated by using the Hansen solubility parameters of PHB and those of the two solvents. The Hansen solubility parameters (*δd*, *δp*, *δh*) for PHB, CYC, and GBL were 15.5, 9.0, 8.6 [27], 17.8, 6.3, 5.1 [28]; and 19.0, 16.6, 7.4 [28], respectively. The solubility radius of PHB in CYC and GBL could then be calculated using the Hansen equation

(2) Rij=[4(δid−δjd)2+(δip−δjp)2+(δih−δjh)2]1/2

where *i* is the solvent and *j* is the polymer. The calculated results were 6.4 for cyclohexanone and 10.4 for GBL. The solubility radius for PHB was 8.5 [27], indicating that CYC was a better solvent and GBL was a poor solvent for PHB.

**Figure 4:**
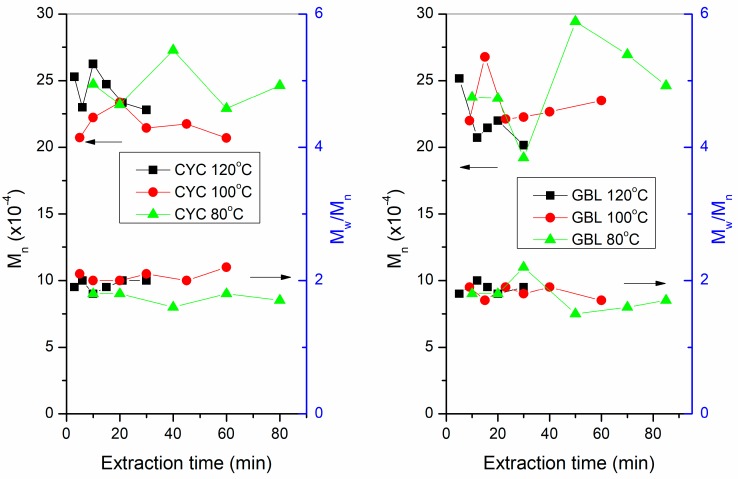
Effect of the extraction solvent cyclohexanone (CYC, **left**) and γ-butyrolactone (GBL, **right**) on molar mass (*M*_n_) and dispersity (*M*_w_/*M*_n_) of the extracted polymers at various temperatures versus extraction time.

**Figure 5:**
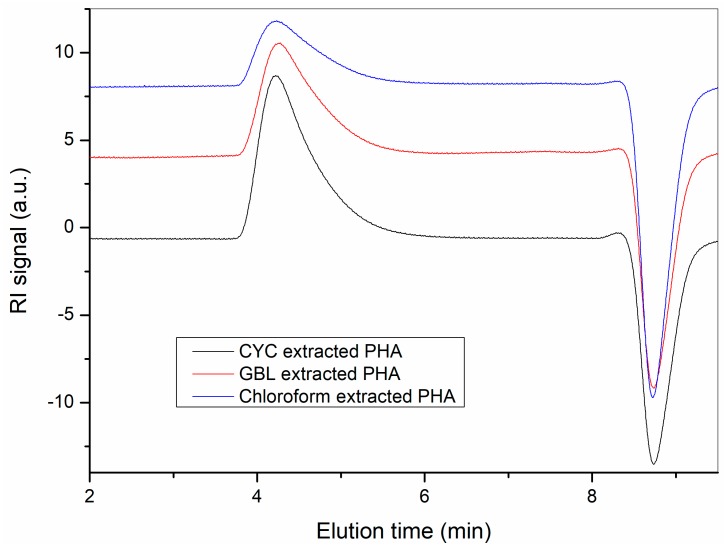
The GPC traces of the PHA extracted using cyclohexanone (CYC), γ-butyrolactone (GBL) at 120 °C for 30 min and chloroform at 60 °C for 24 h. The number average molar mass (*M*_n_) and dispersity (*M*_w_/*M*_n_) of the extracted polymers are 211 kD/2.36, 213 kD/2.23, and 185 kD/2.63, respectively.

The effect of the two extraction solvents on molar mass and dispersity of the extracted polymers is shown in Figure 4. The number average molar mass (*M*n) and dispersity (*M*w/*M*n) of the polymer extracted using chloroform for 24 h was 23 kDa and 2.2, respectively. For both solvents, the dispersity of the extracted polymers had a value of around 2, and the molar mass had an average value of about 23 kDa, regardless of the type of solvent and the temperature and extraction time. Moreover, the molecular weights of the extracted polymers were not significantly affected by extraction time and temperature; this is a considerable benefit when compared to the long-term extraction using halogenated solvents at high temperature where losses of molar mass due to random and chain-end scission of PHA macromolecules have been reported [22]. A comparison of the GPC traces of the PHA extracted using CYC, GBL, and chloroform is shown in Figure 5. There was a longer tail in the GPC trace for the PHA extracted using chloroform, which may indicate a slight degradation of the polymer during extraction using chloroform, while the PHAs extracted using CYC and GBL did not have such tails in their GPC traces.

It should be noted that the amount of polymer extracted from the cells was different. At higher temperatures, more polymers were extracted and less polymer remained inside the cells. Therefore, the molar mass and dispersity of the polymer extracted at higher temperatures represented a larger amount of the polymers originally present in the biomass. Since the present study demonstrated that the molar mass and dispersity did not depend on temperature and solvent, it can be assumed that the extraction occurs uniformly without selectivity, i.e., non-selective extraction of both low and high molar mass PHA chains. Most remarkably, the two ecologically benign, non-halogenated solvents displayed extraction behavior similar to the precarious solvent chloroform.

**Figure 6:**
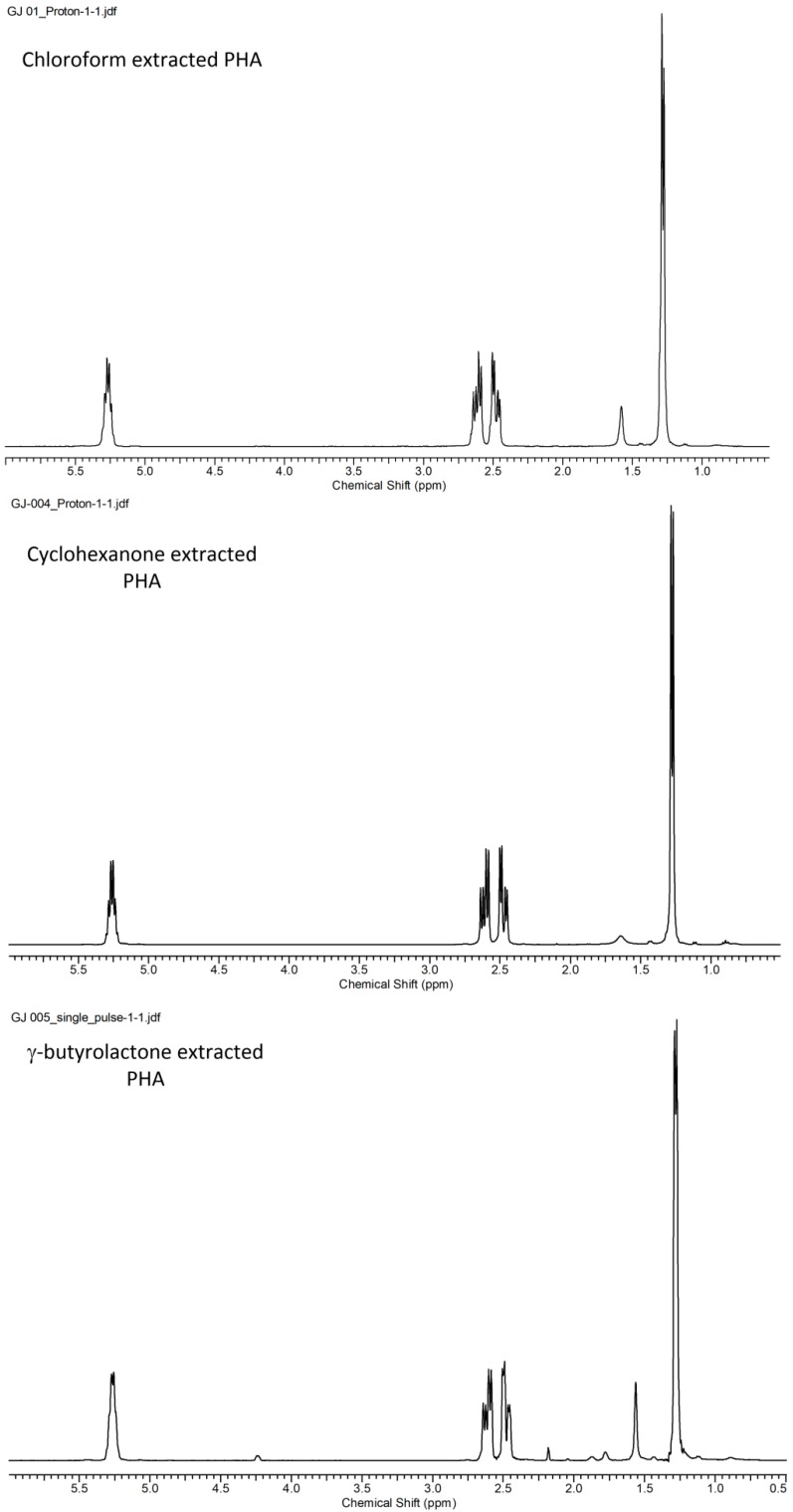
^1^H NMR spectra for the PHA extracted from the biomass using chloroform, CYC, and GBL, respectively.

The ^1^H NMR spectra of the polymers extracted using CYC and GBL, respectively, are shown in Figure 6 in comparison with the polymer extracted using chloroform. It can be seen that the spectra were quite similar for different solvent extractions, indicating that both solvents did not affect the structure of the extracted PHB. The PHB extracted using GBL had a little solvent residue at δ = 2.35 ppm and 4.40 ppm.

Solvent extracted PHAs may contain residual protein and, in the case of Gram-negative cell factories like *C. necator*, high levels of endotoxin, both of them being potent pyrogens that affect the biocompatibility of the produced PHAs by causing inflammatory reactions [29]. The residual protein can be determined using elemental analysis of the nitrogen content in the polymer. Table 1 shows the elemental analysis of the PHA extracted using various solvents.

As can be seen from Table 1, the element nitrogen was not detected for the chloroform extracted polymers. For the polymers extracted using CYC, the average nitrogen content decreased with an increase in extraction temperature, from 0.111 wt % at 80 °C to 0.001 wt % at 120 °C. At the same temperature and extraction time, the nitrogen content of the GBL extracted polymer was 0.003 wt %, which was higher than that extracted using CYC. The nitrogen content in the extracted polymers may be related to the solubility of proteins in the solvents and the variation of solubility with temperature. The nitrogen content of the polymer extracted using CYC at 120 °C was quite similar to the *mcl*-PHA extracted using ethanol [5]. It is interesting to note that the polymer could, in addition to the NMR studies described above, be further verified as PHB according to the carbon to hydrogen ratio based on the elemental analysis listed in Table 1.

For industrial application, several factors have to be considered. Rosengart et al. [21] examined 10 non-halogenated solvents for PHB extraction in terms of waste problems, environmental impact, health, flammability and explosion, reactivity and stability, life cycle score, legislation flag, EHS (environmental, health and safety) flag, boiling point and melting point according to the GSK Solvent Selection Guide. In this study, CYC was demonstrated to be a “good” solvent. Beyond that, we demonstrated its suitability in terms of kinetics of extraction and the effect of extraction time on the molar mass and dispersity of the extracted polymer in the present study. In order to reduce the cost of separation of CYC and the anti-solvent used to precipitate the dissolved polymer, the polymer can be precipitated out of the solution by cooling down the solution to room temperature, at which the solubility of PHA is very low; a similar approach has been previously reported in the case of using acetone as the PHA-solvent [18]. In the CYC case, this can be seen from Figure 3, where the solubility of PHA had a significant drop from 120 to 80 °C.

## 4. Conclusions

Currently, solvent-based recovery of PHAs is still the most effective technique in terms of polymer yield and purity for medical applications. However, the most often used solvents are halogenated, eco-toxic compounds. The harmful character of halogenated solvents is a major drawback of this method when compared with non-solvent based recovery. In this work, both CYC and GBL were examined for their performance in the extraction of PHAs from cells. At 120 °C, the extraction was completed in 3 min with a recovery yield of 95% in the case of CYC, while GBL could only recover 50%. Moreover, both solvents had no effect on the molar mass and dispersity of the extracted polymer. Furthermore, the solubility of PHB in CYC decreased rapidly at low temperatures. This can be used to precipitate the dissolved PHB by cooling down the solution, which can improve the biocompatibility of this biopolyester for medical or cosmetic purposes. Therefore, it can be concluded that the easily recyclable ketone CYC is a potential substitute for halogenated solvents that can provide fast PHA extraction with high purity and recovery yield without compromising the molecular structure and molar mass distribution. Moreover, the described methodology is of potential use for the purification of commercially available PHA (which could contain higher levels of nitrogen), and further studies in this area are currently under way at our laboratories.

It is worth noting that among the commercially produced PHA, aqueous extraction is currently being used [30,31,32,33]. This particularly concerns the promising biodegradable copolymer PHBHHx, whose structure at the molecular level has been already established [34]. Thus, the biocompatibility improvement achieved by the methodology described herein is of potential use for the purification of commercially available PHA (which could contain higher levels of nitrogen), and further studies in this direction are currently under way at our laboratories.

## Figures and Tables

**Table 1:** Elemental analysis of the PHA extracted using non-chlorinated solvents. Two parallel measurements were conducted for each sample.

Extraction Conditions	C (wt %)	H (wt %)	N (wt %)
CYC, 80 °C, 1 h	57.05	6.802	0.113
	56.72	7.099	0.009
CYC, 100 °C, 30 min	57.54	7.160	0.008
	55.60	6.920	0.006
CYC, 120 °C, 10 min	56.05	7.088	0
	55.13	7.096	0.002
GBL, 120 °C, 10 min	56.18	6.894	0.005
	55.29	7.060	0
Chloroform, 61 °C, 24 h	55.28	7.044	0
	55.64	7.033	0
